# Subungual Exostosis of the Hand: A Case Report in a 5-Year-Old Boy and Literature Review

**DOI:** 10.5435/JAAOSGlobal-D-21-00239

**Published:** 2022-03-22

**Authors:** Meagan E. Womack, Olivia J. Fisher, Matthew R. Landrum, Ryan A. Rose

**Affiliations:** From the UT Health San Antonio, San Antonio, TX.

## Abstract

Subungual exostosis is a relatively uncommon benign tumor that occurs at the distal end of the distal phalanx of the toes and rarely the hands. We present in this article a review of the currently published English literature and provide a case report of a 5 year old male with subungual exostosis of the thumb.

A 5 year old male presented with a slow growing mass of the distal dorsal aspect of the left thumb. Radiographs showed dorsal calcifications on the thumb. Surgical removal of the mass and histopathological analysis was performed supporting a diagnosis of subungual exostosis. Post-operatively, the patient had complete excision of the mass, normal nail morphology, no reoccurrence, and no post-surgical complications.

Subungual exostosis remains a rare entity especially in the upper extremity. Its cause is not fully understood, nor is there an agreed upon method of treatment. However, with careful dissection during surgical removal good outcomes can be obtained. To our knowledge, this is the largest literature review on subungual exostosis and our case report is an uncommon presentation in the youngest reported male patient. It is our hope that this literature review and case report lend to increased awareness of subungual exostosis and how to diagnose and treat this lesion.

Subungual exostosis (SE) is a relatively uncommon entity as evidenced by a review of the literature in 1992 revealing only 203 cases of subungual exostosis at that time.^1^ There are even fewer reports of this entity occurring in the upper extremity; only 21 of those 203 cases (10%) were reported in the hand. SE presents most commonly in the adolescent and young adult population with a predilection for the feet.^1,2^ Of these, 70% to 80% occur in the hallux.^3-5^ SE was originally coined as “Dupuytren exostosis” because it was first identified by Dupuytren in 1847 in the hallux.^6^ Hutchinson was the first to describe SE in the upper extremity, specifically its characteristic nail disturbance.^7^ A literature review of the English literature revealed 500 cases of SE with a total of 422 cases affecting the foot and 78 cases in the hand. Because SE is a rare tumor, often patients consult more than one physician or are improperly treated before arrival at a correct diagnosis. The purpose of this article is to present a recent review of the literature and to report a case of SE in a 5-year-old male patient to raise awareness of this uncommon lesion.

## Methods

### Search Strategy

A computerized literature review of PubMed was conducted on March 27, 2021, using the terms “subungual” and “exostosis.”

### Study Selection

A review was performed on articles involving cases of subungual exostosis of the fingers, toes, or a combination of both. Articles covering solely other pathologies, such as osteochondroma or review articles were excluded. Studies published in languages other than English were also excluded. Articles were initially reviewed by their abstracts for inclusion/exclusion criteria, and if the article was unable to be classified by its abstract, it was read in its entirety.

### Data Extraction

Ninety-three articles were found to have lower extremity or upper extremity phalanx involvement or a combination of both. These 93 articles were reviewed for the number of cases and the anatomic location of the SE. Hand case articles were reviewed for the affected digit, age of the patient at presentation, laterality, and sex. In some articles, not all of the variables were documented. These cases were still included in this study, but the statistical evaluation excluded these unknown variables. Some articles included literature reviews of their own with historical articles that were not isolated from the literature search. These historical cases were also included in the dataset. All of the data was stored in an Excel file.

## Results

There were 189 articles identified from the PubMed search. The abstracts and titles of the articles were reviewed for inclusion. Articles that could not be categorized by their abstract, or those that lacked an abstract, were read in their entirety to determine whether they met the inclusion criteria. Of the 189 articles, 43 were not in English and 53 were either unrelated to SE or were review articles and were therefore excluded. After this review, 93 articles were identified from the PubMed search as having cases to review. One of the foot articles listed two other articles' results with toe cases that were not identified through the PubMed search. Carroll et al listed the results of seven articles with finger cases that were not identified through the PubMed search. These unlisted results were then included for a total of 102 articles, with cases for review.

There were a total of 500 cases of subungual exostosis identified from the literature review. Four hundred twenty-two of these cases (84.4%) occurred in the lower extremity and 78 cases (15.6%) in the upper extremity. Excluding unknown variables, the average age of the cases in the hand was 32.3 years with a range from birth to 73 years and an SD of 20 years. There were 33 cases missing information on age. There were 32 females and 12 males creating a female-to-male ratio of 2.7:1. There were 35 cases missing information on sex. The index finger (IF) was the most affected digit. There were 18 thumb (22.8%), 28 IF (35.4%), 17 long finger (21.5%), 8 ring finger (10.1%) , and 8 small finger (10.1%) cases (Tables 1 and 2). There were no missing cases pertaining to the affected digit.

**Table 1:** Breakdown of Digits Affected by Subungual Exostosis in the Hand by Article

Digits affected by Subungual Exostosis							
Article	Age	Sex	Digit	Article	Age	Sex	Digit
Hoehn et al^1^	17 yr	M	L LF	De Berker et al^26^	15 yo	F	LF
Miller-Breslow et al^5^	50 yr	F	L LF		32 yo	F	LF
Li et al^8^	8 yr	F	R IF	Delivanis et al^27^	54 yo	F	R thumb
Kumar et al^9^	7 yr	M	R IF	Carroll et al^3^	69 yo	F	L SF
Starnes et al^10^	15 yr	F	R RF		25 yo	F	L RF
Dave et al^11^	19 yr	F	R thumb		27 yo	F	L LF
Nowillo et al^12^	38 yr	M	R IF		53 yo	M	L IF
Ward et al^13^	65 yr	F	L LF		46 yo	F	L thumb
Pascoal et al^14^	7 yr	Unk	L thumb		29 yo	F	R IF
Oliveria et al^15^	Unk	M	Thumb		34 yo	F	R IF
	Unk	F	Thumb		39 yo	F	R IF
	Unk	F	IF		49 yo	F	R IF
	Unk	F	RF		52 yo	F	R IF
Guidetti et al^16^	47 yr	F	R LF		54 yo	F	R LF
Piccolo et al^17^	32 yr	F	R IF		79 yo	F	R RF
Li, Yizhong et al^2^	Unk	Unk	RF		21 yo	M	R SF
	Unk	Unk	IF		26 yo	M	R SF
Muse et al^18^	24 yr	F	L LF		39 yo	F	R SF
Lee et al^19^	25 yr	F	R LF		44 yo	M	R SF
	29 yr	F	L LF	Tan et al^28^	14 yo	M	R RF
Suga et al^20^	67 yr	F	IF	Hutchinson^7^	Unk	Unk	LF
	73 yr	F	LF	Stocks and Barrington	Unk	Unk	Thumb
Ippolito et al^21^	Unk	Unk	IF		Unk	Unk	Thumb
	Unk	Unk	IF		Unk	Unk	IF
	Unk	Unk	LF		Unk	Unk	IF
Landon et al^22^	Unk	Unk	Thumb		Unk	Unk	IF
	Unk	Unk	Thumb		Unk	Unk	LF
	Unk	Unk	Thumb		Unk	Unk	RF
	Unk	Unk	IF		Unk	Unk	SF
	Unk	Unk	IF		Unk	Unk	SF
Lowenthal et al^23^	4 yr	F	R IF	Mason	Unk	Unk	IF
Iizuka et al^24^	40 yr	F	R LF	Evison and Price^29^	13 yo	M	IF
Matthewson et al^4^	15 yr	Unk	Thumb		34 yo	M	SF
	13 yr	Unk	Thumb	Jacobson	Unk	Unk	Thumb
	13 yr	Unk	Thumb		Unk	Unk	Thumb
	40 yr	Unk	IF	Bennett and Gammer	Unk	Unk	Thumb
	15 yr	Unk	IF	Preaux	Unk	Unk	IF
	Birth	Unk	LF	Baran and Sayag	Unk	Unk	IF
Gӧktay et al^25^	Unk	Unk	R IF				
	Unk	Unk	L RF				

IF = index finger, LF = long finger, RF = ring finger, SF = small finger, Unk = “unknown” variable

**Table 2:** Summary of the Literature Review Results of Subungual Exostosis by Digit Affected

Summary of Digits Affected by Subungual Exostosis	
Digit	No. of Cases
Thumb	18
Index finger	28
Long finger	17
Ring finger	8
Small finger	8

### Case Report

**Figure 1:**
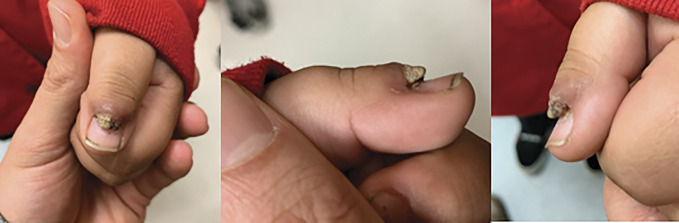
Photographs showing preoperative images of the patient's left thumb.

The patient is a 5-year-old boy without a medical history who presented initially to the pediatric orthopaedic clinic with a chief complaint of a left thumb mass that had been slowly growing for approximately 1 year. He had previously been evaluated by his pediatrician and a dermatologist. The patient was then referred to the orthopaedic hand clinic for an additional evaluation. The mass was mostly nonpainful; however, there was some pain associated with hitting the affected area on other objects accidentally. No associated numbness, tingling, or constitutional symptoms were observed. No recollection of trauma to the digit was observed. Over-the-counter treatment was attempted with wart cream that did not slow the growth of the mass. Examination at the first clinic visit was significant for an exophytic mass to the dorsum of the thumb at the hyponychium (Figure 1). There were no signs of infection; it did not limit his motion, nor was the mass tender to palpation.

### Imaging

**Figure 2:**
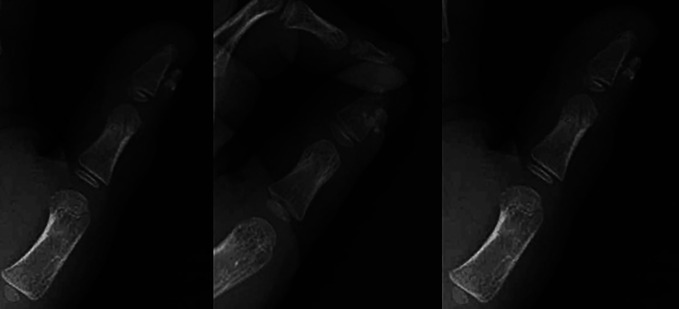
Radiographs showing three views of the left hand demonstrating dorsal calcification overlying the distal phalanx of the thumb.

Radiographs before the first clinic visit showed soft-tissue swelling and calcification over the dorsal distal phalanx of the thumb without connection to the phalanx (Figure 2).

## Surgery

**Figure 3:**
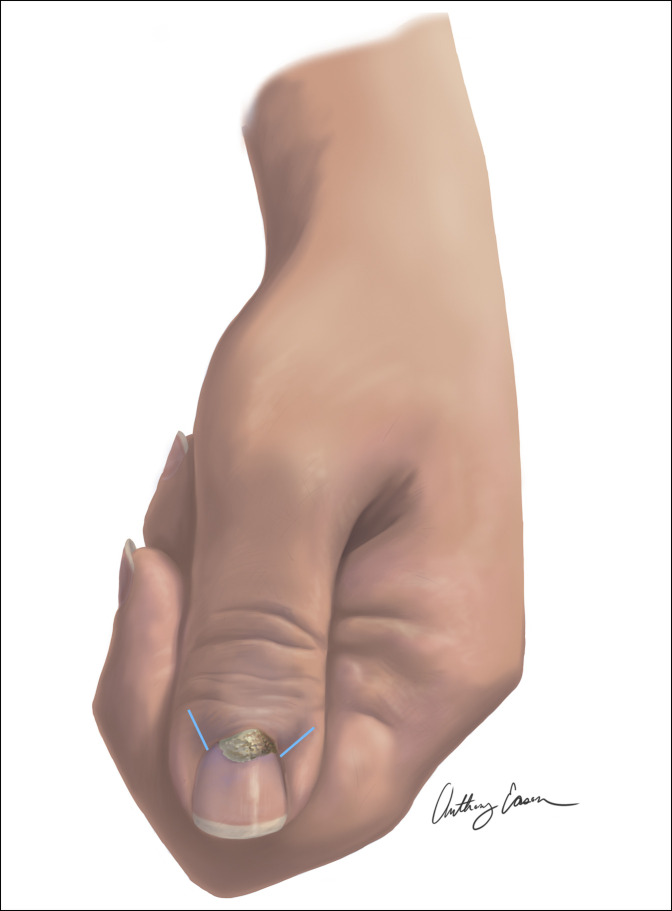
Illustration showing the image of the patient's left thumb with a surgical approach.

The surgical approach consisted of two incisions: one on the dorsal radial aspect of the thumb and the other on the dorsal ulnar aspect of the thumb. The incisions started at the nail fold distally and were carried approximately 1 cm proximally on either side of the thumb (Figure 3). These were used to create a flap at the base of the nail fold. The dissection was then carried down to the nail, and the tumor was not found to be connected to the nail plate. A plane of dissection was isolated, and a Beaver blade was used to separate the mass from the overlying soft tissues. The proximal base of the mass was noted to be extending from the germinal matrix of the digit. Care was taken to minimize the germinal matrix disturbance while fully removing the mass and associated calcifications. The intraoperative margins were negative because the mass, measuring 1.0 × 0.6 × 0.4 cm, was well encapsulated and easy to excise. Intraoperative radiographs were obtained, which identified that the mass and its calcifications had been completely removed.

## Histopathology

**Figure 4:**
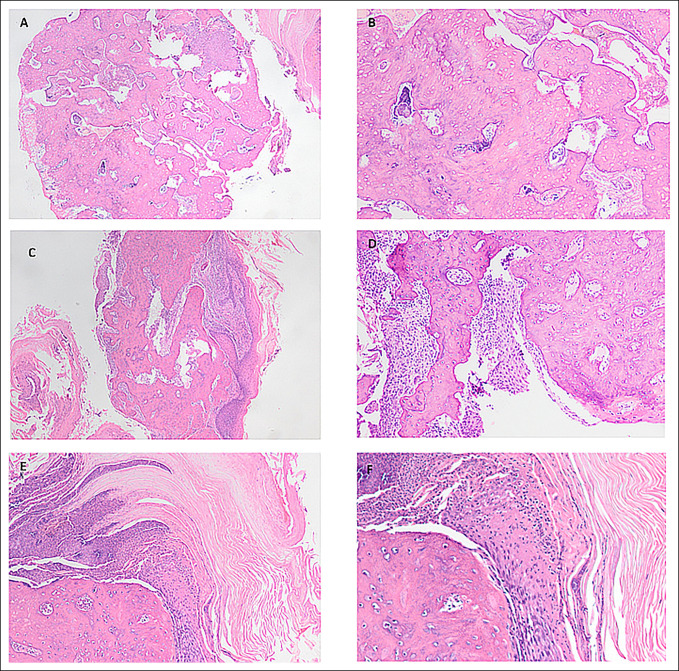
**A**, Hematoxylin and eosin (H&E) stain at ×4 magnification. The image displays an immature, disorganized bony lesion. **B**, H&E stain at ×10 magnification. The image displays the immature, disorganized bone with necrosis. **C**, H&E stain at ×4 magnification. The image displays the superficial bone with inflammatory infiltrates eroding into the squamous layer. **D**, H&E stain at ×10 magnification. The image displays the bone lesion adjacent to squamous tissue, indicating a superficial lesion. **E**, H&E stain at ×10 magnification. A higher power image displaying inflammation overlying the superficial bone with hyperkeratosis. **F**, H&E stain at ×20 magnification. Additional imaging of the superficial bone with overlying inflammation and hyperkeratosis.

The mass was reviewed by a pathologist in association with the musculoskeletal radiology team. The pathology report described the mass as having “florid reactive bone formation.” The microscopic images demonstrated a disorganized, immature bony lesion that could be seen abutting the epidermis. There were areas of necrotic bone with overlying acute inflammation of the epidermis, indicating ulceration. Despite the lack of a cartilaginous cap and no obvious connection to the underlying phalanx, these histopathologic findings are most consistent with subungual exostosis (Figure 4).

## Postoperative Course

**Figure 5:**
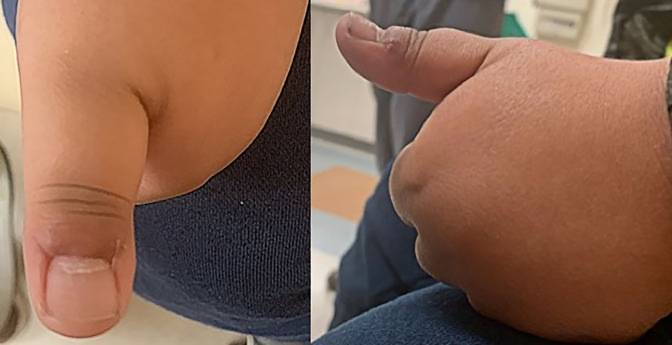
Postoperative images at the 6-week follow-up.

The patient presented for his first postoperative follow-up 2 weeks after surgery and was doing well. He was placed in a stack splint at that time to protect the thumb. At the 6-week follow-up, the patient was without pain, and his motion was full at the interphalangeal joint of the thumb. In addition, the incision had healed fully. There were no signs of infection, and the patient was released to full activity at that time (Figure 5). Unfortunately, after the patient's 6-week follow-up, he did not return to the clinic for additional evaluation for his 6-month follow-up visit. The patient's mother was called at approximately 10 months postoperatively. Per her report, there was no recurrence of the mass, no pain, and no abnormalities in the nail morphology.

## Discussion

SE is a relatively uncommon entity as evidenced by a review of the literature in 1992 revealing 203 cases of SE.^1^ Only 21 of those 203 cases (10%) were reported in the hand. A more recent literature review done in 2013 revealed less than 60 cases occurring in the upper extremity.^13^ This review of the literature reiterates that SE in the upper extremity is uncommon and continues to occur less frequently than SE in the lower extremity. There were 500 cases identified by this literature search. Four hundred twenty-two of those cases (84.4%) occurred in the foot, while only 78 cases (15.6%) occurred in the hand. Because of its rarity, SE tends to be misdiagnosed, leading to improper treatment, as evidenced by our patient's parents' attempt at treating the lesion like a wart.^3^ These lesions have been confused with verruca, onychomycosis, pyogenic granuloma, osteochondroma, or even chondrosarcoma, which can lead to treatment like an infectious process or even like a malignancy.^5,21,29,30^ Often the patients visit more than one physician in different specialties before arrival at the correct diagnosis, as was experienced by our patient.^21,30^

The cause of SE is unknown. The leading hypothesis is that these masses are reactive growths.^29^ The thought is that trauma, infection, or chronic irritation leads to a cartilaginous metaplasia, which then undergoes enchondral^5^ or intramembranous ossification,^21^ leading to bone formation. However, this bone formation from metaplasia has not been associated with malignant transformation.^5^ These masses have been found to have a pathognomonic translocation (X;6), which leads to increased expression of insulin receptor substrate four; however, the effects of this upregulation are not fully understood.^31^ This pathognomonic translocation lends evidence toward a possible hereditary or neoplastic process.

The lesion has been reported to occur more frequently in women than men; however, some reports claimed near equal distribution among sexes.^5,17,29^ This review of the literature revealed that women tend to be more affected than men with a ratio of 2.7:1. Furthermore, this lesion tends to occur in the adolescent to young adult population or in the second and third decades of life.^1,2^ This literature review revealed that the average age of individuals affected by SE in the hand was approximately 32 years. The youngest patient affected by SE was reported at birth; however, this report did not reveal the patient's sex.^4^ The youngest female patient affected by SE was 4 years old,^23^ and before this study, the youngest affected male patient was 7 years old.^9^ Our case report, to the best of our knowledge, is the youngest male patient reported in the literature to date to be affected by SE. In addition, previous reports suggested that the thumb and IF tended to be the most affected digits, and this is supported by our literature review.^3,4^ Here, the IF was the most common digit to be affected with 28 of the 78 cases (35.4%).

SE tends to occur on the dorsal aspect of the digit overlying the distal phalanx.^5,29^ The osseous mass is generally connected to the distal phalanx; however, there have been reports of SE occurring without connection to the distal phalanx.^25,29^ The connection tends to occur away from the epiphyseal line, which helps distinguish SE from an osteochondroma affecting the digit.^29,32^ Furthermore, the mass tends to grow beneath the nail plate and have a connection with the germinal matrix or nail plate, but this is not always true, and some lesions grow upward from the nail bed.^5,15,25^ Radiographs of the lesion help make the diagnosis in conjunction with surgical excision for histopathologic analysis. Radiographs, especially a lateral view, will reveal an osseous structure with connection to the distal phalanx of the digit.^5,25,29^ The periphery of this lesion on imaging may seem hazy. This haziness is due to the cap of tissue overlying the osseous mass.^2,3,5,29^ In most cases, this cap is made up of fibrocartilage, which helps distinguish SE from an osteochondroma.^5,29^ Osteochondromas tend to have a hyaline cartilage cap.^29^ However, some SE cases consist of fibrous tissue caps, whereas others lack a cap altogether.^21,29^ Microscopically, SE tends to lack inflammatory cell infiltration unless there is overlying ulceration of the nail bed.^5^ In our patient, the mass was located dorsally but grew upward from the nail bed similar to the case reported by Goktay et al.^25^ The patient's mass did not show a connection on radiographs to the distal phalanx and did not have a fibrocartilage cap; however, it did have a fibrous cap and inflammatory cell infiltrate secondary to it being a protruded lesion. Finally, our patient's mass did have a connection to the nail bed and germinal matrix, which had to be carefully dissected to preserve nail growth.

The treatment for SE is surgical removal, with the most common complications after removal being onycholysis or recurrence.^5,20,30^ Recurrence is thought to occur when too much of the lesion is left behind, while onycholysis occurs when the nail bed has been overly violated.^5,20-22,30^ There is not an agreed-upon method of removal, although several techniques have been described in the literature. Malkoc et al^30^ described the removal of the nail and an “L” shaped incision through the nail bed with the removal of the mass and suturing the nail bed and nail back down. They did not report any cases of onycholysis or recurrence. Others have reported using different surgical approaches based on the appearance of the lesion.^20,33^ A fish-mouth approach is used for lesions that did not destroy the nail bed, and a direct approach with nail plate removal was used if the lesion involved the nail bed. Basar et al^33^ did not report any recurrences or cosmetic defects with this dichotomous approach to surgical removal. On the other hand, Suga et al^20^ reported that most of their patients who received a direct approach sustained onycholysis postoperatively, while two of their fish-mouth approaches had recurrence of their SE.^20^ De Cambra et al^32^ described a case report in which they performed a direct approach to remove an SE lesion followed by negative pressure therapy to aid in healing the surgical wound. They described no onycholysis or recurrence. In this case report, we also used a more direct approach to the SE lesion, given its protruded position and placement over the nail plate. The patient ended with a good cosmetic result, without onycholysis, and did not have a recurrence of the lesion at their most recent follow-up. However, 6 weeks is a short period for recurrence to occur in a slowly growing mass, and the literature is sparse on the rate of recurrence. However, it was observationally noticed by Miller-Breslow et al^5^ that most of their recurrences occurred within the first year. Given that the patient did not return for a follow-up after 6 weeks, a phone call was made at approximately 10 months post-operatively. Per the mothers report, there was no recurrence and still a good cosmetic result.

In conclusion, SE remains a rare entity, especially in the upper extremity. Its cause is not fully understood, nor is there an agreed-upon method of surgical treatment. However, with careful dissection during surgical removal, good outcomes can be obtained. To the best of our knowledge, this is the largest literature review covering SE, and our case report is an uncommon presentation of SE in the youngest reported male patient. We hope that this literature review and case report lend to increased awareness of SE and how to diagnose and treat this rare lesion.
